# Evaluation of mass treatment with ivermectin program reach and survey coverage for onchocerciasis elimination in selected endemic areas of Ethiopia

**DOI:** 10.1371/journal.pone.0271518

**Published:** 2022-07-28

**Authors:** Gebremedhin Gebrezgabiher, Delenasaw Yewhalaw, Asrat Hailu, Zeleke Mekonnen

**Affiliations:** 1 School of Medical Laboratory Sciences, Institute of Health, Jimma University, Jimma, Ethiopia; 2 College of Veterinary Medicine, Samara University, Samara, Ethiopia; 3 Tropical and Infectious Diseases Research Center, Jimma University, Jimma, Ethiopia; 4 Department of Microbiology, Immunology, and Parasitology, School of Medicine, College of Health Sciences, Addis Ababa University, Addis Ababa, Ethiopia; Kazi Nazrul University, INDIA

## Abstract

Currently, national governments of onchocerciasis endemic African countries are working towards the elimination of the disease using mass drug administration (MDA) with ivermectin as a primary strategy. Attainment of this goal requires implementation of prolonged high MDA coverage in all endemic areas, and vigilant monitoring and evaluation of the program. This study was thus conducted with the purpose of i) providing estimate of ivermectin coverage, ii) validating the MDA coverage reported through community drug distributors (CDDs), iii) determining the factors associated with MDA coverage, and iv) estimating the difference between MDA program reach and survey coverage rates following MDA campaign carried out in May 2017 in Asosa and Yeki districts in Ethiopia. A community-based cross-sectional study was conducted among 2,824 study participants in Asosa and Yeki districts. A total of 50 *kebeles* (smallest administrative units) were randomly selected from the two districts. A systematic sampling was employed to select study households from the 50 *kebeles*. Then, a household member was randomly selected for the interview. Univariate and multivariate logistic regression analysis were used to determine the odds ratio and to observe the associations between the MDA survey coverage and the variables used. Eighty-seven percent (2458/2824) of the respondents from both districts responded that they were offered ivermectin during the May 2017 MDA campaign. At the district level, 1182 individuals from Yeki and 1276 from Asosa, received the drug, that indicate 88.5% and 85.8% MDA program reach in Yeki and Assosa districts, respectively. Whereas, a total of 366 individuals were not offered ivermectin in both study districts. Of these, 47(12.8%), 143(39.1%), and 176(48.1%) did not receive the drug because of program implementation-related reasons, ineligibility criteria, and personal issues, respectively. Of the 1488 and 1336 respondents in Asosa and Yeki, 1272 and 1182 participants took the drug, resulting in survey coverage rate of 85.5% (95% CI: 83.6–87.2%) and 88.5% (95% CI: 86.7–90.1%), respectively. Multivariable logistic regression analysis revealed significantly low survey coverage rate in females (AOR = 0.5, 95%CI: 0.3–0.6; p<0.001) and in those whose age ranges between 15–24 years (AOR = 0.5, 95%CI: 0.3–0.8; p = 0.007) and 25–34 years (AOR = 0.5, 95%CI: 0.3–0.9; p = 0.021) in Asosa. The researchers believe that the current study generated operational evidence on MDA program reach and coverage rates in two study districts in Ethiopia. The survey coverages were lower than the recommended 90% minimum threshold for success. Only Yeki district reached the 90% threshold survey coverage. Both districts had reported higher coverages than the survey estimates (even outside the 95% CI), thus, were not validated. The majority (60.9%) of the reasons for not receiving the drug were related to program implementation and recipients`personal issues. Efforts must therefore be directed to enhance MDA coverage in future rounds via proper MDA planning and implementation, such as allocating adequate time to the MDA activities, health education, and mobilizing of all segments of the population, including adolescents and the youth. The researchers also recommend such studies to be extended to other MDA programs for other neglected tropical diseases (NTDs).

## Introduction

For more than five decades, the World Health Organization (WHO) has led international efforts against onchocerciasis [1], a debilitating dermal and blinding tropical parasitic disease [2]. The initial efforts involved weekly aerial spraying of rivers with larvicides where Simulum vectors were breeding by the former onchocerciasis control program (OCP) in 11 West African countries [3, 4]. The major efforts to control onchocerciasis were upheaved after the introduction of ivermectin (Mectizan®) in 1987 for human use to control onchocerciasis [3, 5–7]. Then, it was quickly complemented with vector control in many areas of the OCP [8, 9] and finally led to the launching of the second WHO-led onchocerciasis control program named African program for onchocerciasis control (APOC) in endemic countries outside the OCP area [4]. The APOC employed mass drug distribution approach commonly called community-directed treatment with ivermectin (CDTi) as its principal strategy to deliver ivermectin to communities of endemic countries in a sustainable way [4, 10, 11].

A recent extensive epidemiological evaluation of onchocerciasis in the late APOC member countries showed that the disease is no more a public health concern in most areas in Africa [12]. Most importantly, the program has successfully interrupted transmission of *Onchocerca volvulus* in some foci in endemic countries [13–22]. Recently, national disease control programs has set a more ambitious goal for the complete elimination of the disease from selected African countries by 2020 [23, 24] and by 2025 [25–27]. Governments of endemic countries have also pledged to achieve the targeted goal under the technical support and oversight of the Expanded Special Project for the Elimination of Neglected Tropical Diseases (ESPEN).

Realization of the goal in endemic areas requires the attainment of complete geographic coverage, consistent high drug coverage [28–30], and robust monitoring and evaluation of the implementation of mass drug administration (MDA) program [31]. Usually, monitoring of MDA coverage is carried out based on routine coverage reports, by compiling and analyzing data obtained from records of community drug distributors (CDDs) during each MDA round [29, 32, 33]. Nonetheless, the CDDs data are usually prone to manipulation and errors [29]. It is, therefore, essential to undertake community-based post-MDA surveys to accurately estimate the drug coverage and validate the reported coverage [28, 29, 33–35].

Onchocerciasis is endemic in 188 districts in Ethiopia, where more than 17 million people are at risk of acquiring infection, and 5.8 million live in highly endemic areas [36]. The CDTi program is the primary strategy employed to control and eliminate the disease since its launch in the Keffa-Sheka Zone of South Nations and Nationalities Peoples Regional (SNNPR) state in 2001 [37]. In 2013, the Federal Ministry of Health (FMOH) had declared that the country`s National Master Plan shifted from onchocerciasis control to the elimination to be achieved by 2020 [38]. Following this strategic shift, the country is currently implementing biannual MDA program in endemic districts. Successively reported national and district level MDA coverage records show a more than 80% coverage in major endemic areas where the MDA is being implemented [39]. However, the accuracy of the reported coverage data are questionable so that it calls for further community-based studyin order to validate and complement these reports. In addition, while the reported coverage is encouraging, the transmission of onchocerciasis has continued in many areas [39], including Yeki district of SNNPR [12, 40] and Asosa of Benishangul-Gumuz region, where the current study was undertaken. Up to now, no study was conducted to identify the problem, for example, to examine if the reported ivermectin coverage in the areas is accurate or not. Moreover, the reported coverage figures made available after every round of MDA did not provide a specific reasons why specific groups in a community did or did not take the drug [41]. Furthermore, the report did not indicate the reasons for not dispensing and receiving the drug and the possible factors impacting the coverage of MDA program.

Previous studies suggested different socio-demographic factors such as age, gender, ethnicity, and length of stay or residence in their communities as influencing factors of the MDA coverage of control programs in other onchocerciasis endemic countries [42–45]. It is thus relevant to assess and monitor reasons for not offering ivermectin, and explore the factors associated with drug coverage in the MDA program [46]. Besides, though the CDTi program for onchocerciasis is a directly observed treatment (DOT) strategy, the CDDs may not adhere to the treatment guidelines. Hence, either individuals in endemic communities may not receive and take the drug directly from the hand of the CDDs or the CDDs may not supervise/follow them to take it in at the spot. The existence of variation between MDA Program reach and survey coverage rate indicates that eligible members of communities have not swallowed the drug they received so that they are left untreated. This makes them reservoir of parasite and ultimately impede the targeted elimination goal of the disease. This community-based study was, therefore, conducted in the two onchocerciasis endemic districts of the two regions in Ethiopia, namely Asosa, in Benishangul-Gumuzand Yeki in SNNPR to (i) Validate the reported coverage and provide estimate of ivermectin MDA coverage, (ii) Identify the reasons for not receiving ivermectin, (iii) Identify the reasons for not taking the drug offered if that was the case, and (iv) Estimate the differences between MDA program reach and survey coverage rates, and (v) determine factors associated with ivermectin coverage during the May 2017 MDA campaign.

## Methods

### Study setting

A community-based cross-sectional study was conducted in two onchocerciasis endemic districts of Ethiopia namely Asosa of Benishangual-Gumuz region (June to mid-August 2017) and Yeki of SNNPR (from third week of October to the end of November 2017). Detailed description of the study areas is available elsewhere [47].

#### MDA implementation and process

In Yeki, mass ivermectin treatment was launched in 2001 and was running till 2014 on an annual basis with the support of WHO/APOC and The Carter Centre (TCC). The biannual MDA was adopted in 2015 as part of the strategy to facilitate the ultimate interruption of the infection. Now, the program is running in the district with the support of the FMoH and TCC. Till 2014, ivermectin had been distributed via male CDDs. However, after the launching of the biannual mass ivermectin distribution in 2015, the MDA process is implemented via the health development army (HDAs), a team of community volunteer females who were trained to provide neglected tropical diseases (NTDs) services including mass ivermectin distribution for onchocerciasis. The health extension workers (HEWs) are responsible for orienting and overseeing the activity of the HDAs, keeping ivermectin treatment records at *kebele*(the smallest administrative unit) level, and reporting MDA data to district health officials.

Asosa is one of the districts endemic for onchocerciasis in BG region where the MDA program has been running since 2013. Ivermectin is co-distributed with albendazole for Lymphatic filariasis in the district with the support of the USAID-ENVISION project through Research Triangle Institute (RTI) international. As to Yeki district of SNNPR, drug distribution is carried out by the HDA under the supervision and technical assistance of HEWs. However, most of the HDAs had low literacy skills to keep distribution records of both drugs so the HEWs were responsible to conduct most of the MDA activities. The HDAs assist the HEWs in community sensitization, measuring community members’ height, and providing water when community members swallow the drug.

To implement the MDA program, a cascade of refresher training is given in every round of the drug distribution in Yeki and Asosa. An orientation or refresher training is provided to HEWs before the drug distribution at their respective districts. The HEWs in turn train the HDAs and the community leaders to sensitize and aware them about onchocerciasis, including how to organize the MDA. At the community level, training involves health education of each community about the disease and its control. The training is followed by intense community mobilization and sensitization using posters and messages delivered by village leaders and HDAs to inform and encourage the community to participate in the MDA campaign. This is conducted in each village using mechanisms such as social and religious gatherings. The HDAs then distribute the drug to community members. Following the drug distribution, coverage data is collected from each drug distribution sites and reported to the district health office.

### Source population

All people living in the communities of the selected districts during the study period were used as source population.

### Study population

All community members aged five years and above during the 2017 MDA year were considered as study population.

### Inclusion criteria

All community members aged five years and above during the 2017 MDA year living in the study areas were included in the study.

### Exclusion criteria

Those aged below five years, terminally ill, or with a mental health problem that makes the interview difficult, were excluded. Moreover, non-residents who came from other places after the May 2017 MDA campaign were excluded from the study.

### Sample size calculation

The sample size (n) was calculated using the following formula [29]:

$$n=\frac{\left(DEFF)(z_{\alpha /2}^{2})(p)(1-p\right)}{\delta^{2}(1-r)}$$

Where n: survey sample size; DEFF: design effect; α: alpha; δ: desired precision; r: non-response rate, and p: reported drug coverage.

For the last MDA in 2017, the reported drug coverages in Asosa and Yeki were 82% and 88%, respectively. To ensure that the sample size is sufficient, 15% from the reported coverage was subtracted [29]. The sample size is thus planned to estimate a reported coverage of 73% in Yeki and 67% in Asosa. Considering a design effect of four and a non-response rate of 10% with 5% precision at 95% confidence interval (CI), a sample of 1,346 individuals from Yeki and 1,510 from Asosa was estimated. Overall, a sample size of 2,856 participants were estimated.

### Selection of *Kebeles* and study participants

Asosa and Yeki districts have 74 and 22 *kebele*s, respectively. A total of 50 *kebele*s were randomly chosen, 30 *kebele*s from Asosa and 20 *kebele*s from Yeki. The calculated sample size was proportionally allocated according to the household size of the *kebeles*. A systematic sampling of households where sampling interval was employed by dividing the total number of households in each *kebele* by sample allocated for each *kebele*. Larger *kebeles* were first divided into reasonable segments and the sample size allocated to that *kebele* was proportionally re-allocated to the segments. All eligible household members were listed and one member of the household was randomly selected to respond to the interview. In the absence of the selected member, another member from the same household was selected randomly and replaced. Data for young children were collected from their primary caretakers or the children themselves. Houses that were found closed on the day of the survey and heads of households who declined to participate in the study were not replaced.

### Data collection methods

Initially, the *kebele* leaders and HEWs were approached and informed about the purpose of the survey and fixed the day of the week for data collection so that the survey population were sensitized to stay at home. Before starting the interview, all members of a household who were present in their home were gathered; the purpose of the survey was explained, and permission to proceed was sought from the household head. Then, one member each of the household was randomly selected to participate in the face-to-face interviews and the socio-demographic information, household characteristics, and other relevant data were collected to address the stated objectives. The respondents were shown examples of ivermectin tablet to enhance recall.

### Statistical analysis

Data collected through questionnaires were coded and entered into MS excel, and analyzed using the RStudio version 3.2.3 software. Frequencies and proportions were used for the descriptive analysis. The ivermectin MDA program reach was calculated as the proportion of individuals in the survey areas who were offered the drug among the total number of individuals surveyed, regardless of whether it was swallowed or not. The survey coverage rate was calculated as the proportion of individuals in the survey areas who reported swallowing the drug among the total number of individuals surveyed. The reported coverage data are considered validated if they fall within 95% CI of the survey coverage rate. The reported coverage data and population estimates from the MDA were provided by the Onchocerciasis/NTDs focal person of the respective district health office to compare the reported coverage with the survey coverage rate. To compare the surveyed coverage, the researchers used the reported program coverage data among the eligible population (i.e., by subtracting the number of under-five children from the denominator). Univariate and multivariate logistic regression analysis were used to determine the odds ratio and to observe the associations between the MDA survey coverage and the variables used. P-value less than 0.05 (p<0.05) was considered statistically significant.

### Ethical considerations

The study obtained ethical approval from Jimma University Institutional Review Board (IRB; reference number: RPGC/170/06). Permissions were obtained from the Regional/Zonal Health Bureau, District Health Bureau Offices, and *Kebele* administrations. The aim of the study was explained to the study participants, and written informed consent was obtained for those age 18 years and above, or from parents or guardians for participants less than 18 years. The respondents were informed that participation is voluntary, and they were free to withdraw from the study at any time. The data collected from the study respondent were kept confidential.

## Results

### Characteristics of survey respondents

Of the estimated 2,856 participants, 2,824 participated in this study, giving a response rate of 98.9%. And of all those who participated, 1,488 (52.7%) were from Asosa district where ivermectin had been co-administered with albendazole in an integrated approach, while 1,336(47.3%) were from Yeki district. The proportion of male participants was almost equal to the female participants in both districts (51.2% female and 48.8% male in Asosa; 50.4% female and 49.6% male in Yeki). The median ages of the survey respondents were 29 years in Asosa and 28 years in Yeki. The majority of the respondents were from Amara ethnic group in both districts (53.6% from Asosa and 39.5% from Yeki). Table 1 depicts the socio-demographic profiles of the survey respondents in the study districts.

**Table 1:** Socio-demographic profiles of the respondents in Asosa and Yeki districts (2017).

Characteristics		Asosa (n = 1488)	Yeki (n = 1336)	Total (N = 2824)
Gender	Female	762(51.2)	674(50.4)	1436(50.8)
	Male	726(48.8)	662(49.6)	1388(49.2)
Age in years	5–14	208(14)	256(19.2)	464(16.4)
	15–24	404(27.2)	239(17.9)	643(22.7)
	25–34	265(17.8)	318(23.8)	583(20.6)
	35–44	201(13.5)	234(17.5)	435(15.4)
	45–54	175(11.8)	150(11.2)	325(11.5)
	>54	235(15.8)	139(10.4)	374(13.2)
Religion	Evangelical Christian	-	521(39)	521(18.5)
	Orthodox Christian	433(29.1)	484(36.2)	917(32.5)
	Muslim	1055(70.9)	331(24.8)	1386(49)
Ethnicity	Amara	797(53.6)	528(39.5)	1325(46.9)
	Bench	-	74(5.5)	74(2.6)
	Berta	676 (45.4)	-	676 (23.9)
	Kafficho	-	222(16.6)	222(7.9)
	Majang	-	91(6.8)	91(3.2)
	Manja	-	122(9.1)	122(4.3)
	Oromo	3 (0.2)	115 (8.6)	118 (4.2)
	Shakicho	-	87(6.5)	87(3.1)
	Sheko	-	89(6.7)	89(3.2)
	Others	12(0.8)	8(0.6)	20(0.7)
Household size	<5	876(58.9)	933(69.8)	1809(64.1)
	5–10	553(37.2)	387(29)	940(33.3)
	≥11	59(4)	16(1.2)	75(2.6)

Others: Agew, Dawro, Gurage, Hadiya, Menit, Tigray, Wolaita, Yem

### MDA program reach and reasons for not offering/receiving ivermectin during the 2017 MDA campaign

Of the 2824 respondents, 2458(87%, 95% CI: 85.8–88.2%) reported that they received ivermectin during the May 2017 MDA campaign. At district level, 1182 and 1276 respondents were offered the drug in Yeki and Asosa, respectively, indicating the MDA program reach of 88.5% (95% CI: 86.7–90.1%) in Yeki and 85.8% (95% CI: 83.9–87.4%) in Asosa.

A total of 366 individuals were not offered ivermectin in the May 2017 MDA campaign in both study areas. Various reasons were reported for not being offered. The reasons in general fall into three major categories namely program-implementation related issues, personal reasons, and ineligibility criteria, that account for 12.8% (47/366), 48.1% (176/366), and 39.1% (143/366), respectively. Table 2 below presents summary of the reasons.

**Table 2:** Reasons for not offering/receiving ivermectin in Asosa and Yeki districts during May 2017 MDA campaign.

Reasons for not being offered		Asosa	Yeki	Total
		n(%, 95%CI)	n(%, 95%CI)	n(%, 95%CI)
(i)	Didnot hear about MDA campaign	5(2.4%,0.9–5.7%)	23(14.9%,9.9–21.8%)	28(7.7%,5.2–10.9%)
	Drug stock out	9(4.3%, 2.1–8.2%)	5(3.3%,1.2–7.8%)	14(3.8%, 2.2–6.5%)
	HDA/CDD didnot come home	2(0.9%,0.2–3.7%)	1(0.6%,0.03–4.1%)	3(0.8%, 0.2–2.5%)
	Extremely aged	0(0%, 0–2.2%)	2(1.2%, 0.2–5.1%)	2(0.6%, 0.1–2.2%)
(ii)	Absent during the MDA campaign	81(38.2%,31.7–45.1%)	60(38.9%,31.3–47.2%)	141(38.5%, 33.6–43.7%)
	Refused because donot take modern medicine	1(0.5%,0.02–3%)	2(1.2%, 0.2–5.1%)	3(0.8%, 0.2–2.5%)
	Refused because do not take tablet medicine	1(0.5%, 0.02–3%)	0(0%,0–3%)	1(0.3%,0.01–1.8%)
	Refused because taking other medicine	1(0.5%, 0.02–3%)	6(3.9%, 1.6–8.7%)	7(1.9%,0.8–4.1%)
	Refused because of fear of drug side effect	9(4.3%,2.1–8.2%)	8(5.2%,2.4–10.3%)	17(4.6%, 2.8–7.5%)
	Refused because being healthy	6(2.8%, 1.1–6.3%)	1(0.6%,0.03–4.1%)	7(1.9%,0.8–4.1%)
(iii)	Severely sick	17(8%,4.9–12.7%)	5(3.3%,1.2–7.8%)	22(6%, 4–9%)
	Underage	3(1.4%,0.4–4.4%)	9(5.8%, 2.9–11.1%)	12(3.3%, 1.8–5.8%)
	Breast feeding	6(2.8%,1.1–6.3%)	2(1.2%, 0.2–5.1%)	8(2.2%, 1–4.4%)
	Pregnant	71(33.5%,27.3–40.3%)	30(19.4%, 13.7–26.8%)	101(27.6%, 23.1–32.5%)

CDD: Community drug distributor; HDA: Health development army; MDA: Mass drug administration; (i) Program implementation related reasons; (ii) Personal reasons; (iii) ineligibility criteria

### Survey coverage and reasons for not taking ivermectin during the May 2017 MDA campaign

Of the total 2824 respondents from both study districts, 2454 people took the drug. And at the district level, 1272 interviewees, out of 1488 respondents, swallowed the drug in Asosa, resulting in a survey coverage rate of 85.5% (95% CI: 83.6–87.2%). In Yeki, too, of the 1336 respondents, 1182 swallowed the drug revealing a survey coverage rate of 88.5% (95% CI: 86.7–90.1%). In addition, of those who were offered in Asosa, 99.7% (1272/1276) of them swallowed the drug. Four respondents in Asosa, however, did not swallow the drug they were offered. Two of these four respondents reasoned out that they did not swallow the drug for fear of the adverse side effects that follow swallowing of the drug. The remaining two respondents stated taking another drug for another illness and providing the drug to another family member who was absent during the drug distribution time. In comparison, all the 1182 participants in Yeki who were offered the drug had swallowed it.

**Fig 1:**
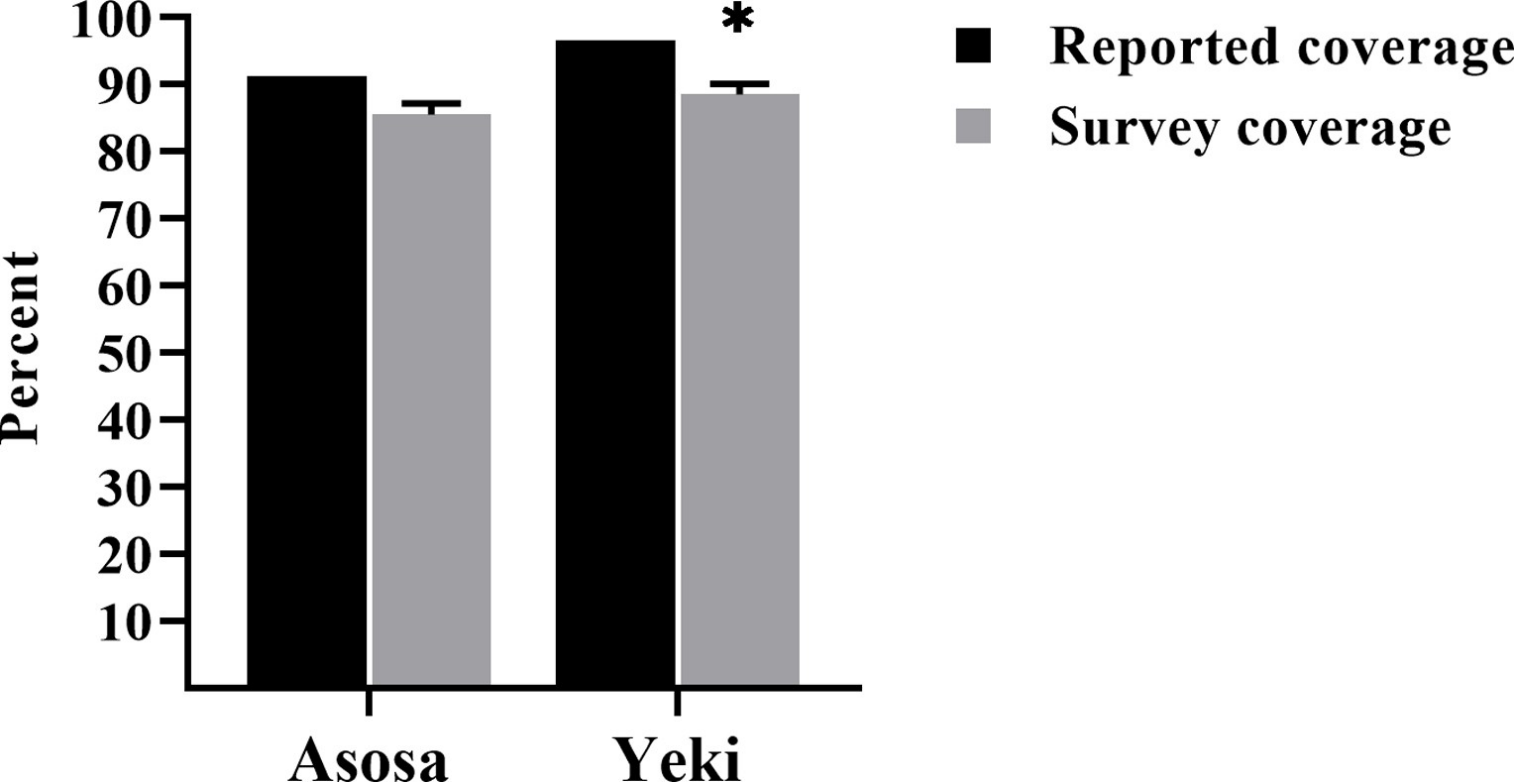
Comparison of reported and survey coverage rate in May MDA campaign in Asosa and Yeki districts. * indicates the survey coverage in Yeki reached the 90% threshold success in its upper 95% confidence bound.

The survey coverage rate was lower than the reported coverage from both districts in the MDA campaign as depicted in Fig 1. The difference was 5.8% in Asosa and 8% in Yeki.

### Factors associated with survey coverage of the May 2017 MDA campaign

Socio-demographic predictors of ivermectin intake were analyzed using univariate and multivariate logistic regression analyses. Accordingly, significantly low MDA coverage rates were observed in the age groups of 15–24 years (COR = 0.4, 95%CI: 0.2–0.7; p<0.001) and 25–34 years (COR = 0.4, 95%CI: 0.2–0.6; p<0.001) compared to the study respondents in the age range of above 54 years. Similarly, female respondents (COR = 0.4, 95%CI: 0.3–0.6; p<0.001), Muslims (COR = 0.6, 95%CI: 0.4–0.9; p = 0.007), and Berta ethnic group (COR = 0.6, 95%CI: 0.4–0.8; p<0.001) had significantly lower survey coverage rates compared to their respective counterparts (Table 3). Among ethnic groups present in Yeki district, the Majang ethnicity had low coverage rate (COR = 0.6, 95%CI: 0.3–0.9; p = 0.049) (Table 4).

**Table 3:** Results of the regression analysis in the MDA coverage in Asosa district.

Variable		Number of participants	Swallowed ivermectin		COR (95% CI)	p-value	AOR (95% CI)	p-value
			No	Yes			
Gender	Female	762(51.2)	147(19.3)	615(80.7)	0.4 (0.3–0.6)	<0.001^†^	0.5(0.3–0.6)	<0.001^†^
	Male	726(48.8)	69(9.5)	657(90.5)	Reference		Reference	
Age in years	5–14	208(14)	24(11.5)	184(88.5)	0.7(0.4–1.3)	0.289	0.7(0.6–2.7)	0.585
	15–24	404(27.2)	78(19.3)	326(80.7)	0.4(0.2–0.7)	<0.001^†^	0.5(0.3–0.8)	0.007^†^
	25–34	265(17.8)	55(20.8)	210(79.2)	0.4(0.2–0.6)	<0.001^†^	0.5(0.3–0.9)	0.021^†^
	35–44	201(13.5)	23(11.4)	178(88.6)	0.7(0.4–1.4)	0.308	0.9(0.5–1.7)	0.693
	45–54	175(11.8)	16(9.1)	159(90.9)	0.9(0.5–1.8)	0.823	1.1(0.6–2.2)	0.758
	>54	235(15.8)	20(8.5)	215(91.5)	Reference		Reference	
Religion	Muslim	1055(70.9)	170(16.1)	885(83.9)	0.6 (0.4–0.9)	0.007^†^	0.9 (0.6–1.4)	0.678
	Christian	433(29.1)	46(10.6)	387(89.4)	Reference		Reference	
Years of stay	≤ 9	75(5)	12(16)	63(84)	Reference		Reference	
	≥10	1413(95)	204(14.4)	1209(85.6)	1.1 (0.6–2.1)	0.708	2.3(0.9–5.4)	0.063
Ethnicity	Amara	797(53.6)	92(11.5)	705(88.5)	Reference		Reference	
	Berta	676 (45.4)	123(18.2)	553(81.8)	0.6 (0.4–0.8)	<0.001^†^	0.7(0.5–1)	0.059
	Other^a^	15(1)	1(6.7)	14(93.3)	1.8 (0.2–14.1)	0.563	2.1(0.3–16.3)	0.49

^a^Agew, Oromo, Tigray, Wolaita;

^†^indicate statistically significant

**Table 4:** Results of the regression analysis in the MDA coverage in Yeki district.

Variable		Number of participants	Swallowed ivermectin		COR (95% CI)	p-value	AOR (95% CI)	p-value
			No	Yes				
Gender	Female	674(50.4)	88(13.1)	586(86.9)	0.7(0.5–1)	0.078	0.8(0.5–1.1)	0.172
	Male	662(49.6)	66(10)	596(90)	Reference		Reference	
Age in years	5–14	256(19.2)	25(9.8)	231(90.2)	1.4 (0.7–2.6)	0.333	1.7 (0.8–3.7)	0.168
	15–24	239(17.9)	35(14.6)	204(85.4)	0.9 (0.5–1.6)	0.647	0.9 (0.5–1.7)	0.712
	25–34	318(23.8)	43(13.5)	275(86.5)	0.9 (0.5–1.7)	0.869	0.9 (0.5–1.8)	0.963
	35–44	234(17.5)	17(7.3)	217(92.7)	1.9 (0.9–3.8)	0.072	1.9 (0.9–3.9)	0.073
	45–54	150(11.2)	16(10.7)	134(89.3)	1.2 (0.6–2.60	0.548	1.2 (0.6–2.6)	0.553
	>54	139(10.4)	18(12.9)	121(87.1)	Reference		Reference	
Religion	Muslim	331(24.8)	33(10)	298(90)	Reference		Reference	
	Christian	1005(75.2)	121(12)	884(88)	1.2(0.8–1.9)	0.307	0.8(0.5–1.3)	0.353
Length of stay	≤ 9	103(7.7)	13(12.6)	90(87.4)	Reference		Reference	
	≥10	1233(92.3)	141(11.4)	1092(88.6)	1.1(0.6–2.1)	0.717	1.6(0.7–3.7)	0.245
Ethnicity	Amara	528(39.5)	64(12.1)	464(87.9)	Reference		Reference	
	Oromo	115 (8.6)	11(9.6)	104(90.4)	1.3 (0.7–2.6)	0.44	1.4 (0.7–2.9)	0.31
	Kafficho	222(16.6)	19(8.6)	203(91.4)	1.5 (0.9–2.5)	0.158	1.5 (0.9–2.6)	0.141
	Shakicho	87(6.5)	7(8)	80(92)	1.6 (0.7–3.6)	0.274	1.7 (0.8–4.1)	0.194
	Sheko	89(6.7)	15(16.9)	74(83.1)	0.7(0.4–1.3)	0.219	0.8 (0.4–1.5)	0.415
	Majang	91(6.8)	18(19.8)	73(80.2)	0.6 (0.3–0.9)	0.049^†^	0.7 (0.4–1.3)	0.212
	Manja	122(9.1)	12(9.8)	110(90.2)	1.3 (0.7–2.4)	0.48	1.5 (0.7–2.9)	0.262
	Other^b^	82(6.1)	8(9.8)	74(90.2)	1.3 (0.6–2.8)	0.538	1.4 (0.6–3.2)	0.376

^b^Bench, Dawro, Gurage, Hadiya, Menit, Yem, Tigray, Wolaita;

^†^ indicate statistically significant

The multivariable logistic regression model estimated that the respondents in the age range of 15–24 years (AOR = 0.5, 95%CI: 0.3–0.8; p = 0.007) and 25–34 years (AOR = 0.5, 95%CI: 0.3–0.9; p = 0.021) had significantly low coverage rates in Asosa. Similarly, a significantly lower coverage rate was observed in females respondents than males in the same district (AOR = 0.5, 95%CI: 0.3–0.6; p<0.001) (Table 3). Years of stay showed no association with ivermectin coverage rate in both districts (Tables 3 and 4).

## Discussion

Elimination of onchocerciasis via mass ivermectin distribution is contingent upon attaining high drug coverage and maintaining it for a long period of time in order to reduce the reservoir of the parasite in humans and the onwards interruption of the transmission of *O*. *volvulus*. Nevertheless, this has been a challenge in MDA programs of onchocerciasis endemic countries [48]. This study was conducted to estimate ivermectin coverage, validate MDA coverage reported through the CDDs, explore the major reasons for not offering and swallowing ivermectin if those were the cases, and determine factors influencing drug intake in the MDA campaign in May 2017 in two onchocerciasis endemic districts, Yeki, SNNPR and Assosa, Benishangul-Gumuz region of Ethiopia.

In agreement with a recent coverage evaluation study conducted in Ethiopia [49] the survey coverage rates were 85.5% (95% CI: 83.6–87.2%) and 88.5% (95% CI: 86.7–90.1%) of the eligible population in Asosa and Yeki districts, respectively. Conversely, the current survey coverage rates were higher than the survey coverage rate reported from Foumbot and Massangam health districts inCameroon [45] and Abia state in Nigeria [50]. It is recommended that the surveyed ivermectin coverage need to reach at least 90% of the eligible population, which is equivalent to 80% of the total population, in all MDA implementation areas to ensure successful interruption of the transmission of *O*. *volvulus* infection [35, 37]. In Asosa, the survey coverage was slightly lower than the 90% recommended minimum threshold for interruption of *O*. *volvulus* infection. Thus, all necessary efforts must be undertaken to enhance coverage in the future MDA rounds. Interestingly, the survey coverage in Yeki reached the 90% threshold success in its 95% confidence limits, and this needs to be continued in a strengthened manner till the targeted goal is attained. Similar to a previous study [45], both districts reported drug coverages higher than the survey estimates and were outside the 95% confidence bounds, indicating the reports were not fully validated. This might be due to inaccurate census data or any influence to achieve the program goals that may cause intentional inflation of the reported coverage [30].

Coverage surveys have helped identify reasons that influence participation in MDA campaigns against NTDs [51–55] including onchocerciasis [35, 49, 50] to implement tailored effective strategies to improve the program. Among the 2824 respondents from both districts, 366 individuals were not offered the drug in May 2017’s drug distribution campaign, i.e., 212 and 154 persons from Asosa and Yeki districts, respectively. Most individuals who were not offered treatment reported that they were not present in their houses and/or community during the campaign, and this counts to 38.9% (60/154) and 38.2% (81/212) of them in Yeki and Asosa districts, respectively. This might be due to the insufficiently allocated time allocated for the MDA campaign that lasted only for a few days for mobilizing, educating and distributing the drug to the communities of the study areas. This eventually resulted in failure to offer the medication to all eligible in need [42]. Consistent with findings of the current study, participants’ absenteeism from their respective homes or village at the time of drug distribution had been identified as a major reason for missing the drug [42–44, 49, 56, 57]. In Yeki, though present in their homes and community, 14.9% (23/154) of the ivermectin non-recipients reported that they lack information about the MDA campaign. It is, therefore, necessary to minimize the levels of non-recipients by allocating a sufficient number of days for the MDA campaign and undertaking effective mobilization of communities preceding drug distribution [58]. HDAs/HEWs and other responsible bodies need to be sure that all eligible members in their communities have adequate information about the schedule of the mass ivermectin distribution in the upcoming MDA rounds. Another important reason that affected the MDA in the study areas is the magnitude of refusals during the campaign: 8.5% (18/212) and 11% (17/154) of the non-recipients refused treatment in Asosa and Yeki, respectively. Fifty percent (9/18) and 47.1% (8/17) of the refusals were attributed to the fear of adverse reactions that occurred after taking ivermectin in Asosa and Yeki, respectively. Communities should be provided with health education regarding the risk of acquiring infection with *O*. *volvulus*, benefits of being treated, and the possible occurrence of adverse reactions [59]. Also, the HDAs/HEWs need to be equipped with antihistamines to manage the cases of adverse drug reactions [60].

Understanding factors associated with low coverage is helpful to improve MDA program implementation. Analysis of factors presumed to impact MDA coverage showed that the female gender was significantly associated with lower coverage rates than their male counterparts in Asosa (p<0.001). This observation might be due to the manufacturer’s guideline, which excluded pregnant and breastfeeding women from taking the drug [61, 62]. However, a finding from another country showed that the survey coverage rates were higher for females than the males [63]. This variation might be due to differences in perceived benefits of treatment, health-seeking behavior, and socio-cultural belief. Age aggregated findings of the study showed that respondents in the age range of15-24 years and 25–34 years had significantly low coverage rates compared to those aged above 54 years in Asosa. This is interesting, perhaps, as those age groups (i) are less likely to have suffered onchocerciasis-attributed morbidity so that they might no longer consider the disease as a threat in their community, (ii) could have poor attendance at village meetings on the benefits of being treated with ivermectin, (iii) are the mobile portion of the population that travel from place to place for several reasons: farming, small-scale gold mining, trading, and attending high school and higher education far from their residential area. Such problems could be avoided through better community education and inclusion of adolescents and the youths in village meetings and community health education programs. This is however contrary to another finding from Uganda that reported that young adults and middle-aged were more likely to take ivermectin than their elderly neighbors [48]. Though the difference was not statistically significant, a lower survey coverage rate was observed in females and 15–24 and 25–34 years age ranges in Yeki district.

By comparing the program reach to the survey coverage rate, it is possible to see the percentage difference between the proportion of individuals who were offered the drug and those who swallow it and see the level of compliance to CDDs program treatment guideline. A large gap between MDA program reach and survey coverage indicates that a large proportion of the target population are not swallowing the drug offered, which is a significant problem in MDA programs of other NTDs [64, 65], including MDA program for onchocerciasis [50]. Interestingly, nearly all participants of the present study who were offered the drug swallowed it, revealing no considerable variation between MDA Program reach and survey coverage rate; only 0.3% of those offered in Asosa did not swallow the drug. Similar findings were reported in other recent studies conducted in Ethiopia [49].

## Conclusions

The present study generated operational evidence on MDA program reach and coverage rates in Asosa and Yeki districts where the MDA program is running as part of the nationwide efforts to interrupt transmission of the infection. The survey coverages were lower than the recommended 90% minimum threshold for success. Only Yeki district included the 90% threshold in its upper 95% CI survey coverage. Both districts reported higher coverages than the survey estimates (even outside the 95% CI). Thus, the validity of these reports are questionable. The main reasons for not offering the drug wererelated to the program and the recipients. The present researchers, therefore, recommend that necessary efforts must be undertaken to enhance MDA coverage in future MDA rounds, such as proper planning and MDA implementation that includes allocating adequate time to the MDA activities, health education, and mobilization of all population segments, including adolescents and young adults. The researchers also recommend such studies to be extended to other MDA programs for other NTDs.

## Limitations of the study

This study relied on the self-report data of household members’ participation in the MDA campaign against onchocerciasis in May 2017. The accuracy of the responses therefore depends on the study respondents’ ability to recall so that the study may not be free of recall bias.

## Supporting information

S1 Table English version interview guide. This is the English version interview guide that was used to collect the socio-demographic profile of the study participants and other relevant data to evaluate mass treatment with ivermectin program reach and survey coverage for onchocerciasis elimination in selected endemic areas of Ethiopia.
(DOCX)

S2 Table Amharic version interview guide. This is the Amharic translated version of interview guide that was used to collect the socio-demographic profile of the study participants and other relevant data to evaluate mass treatment with ivermectin program reach and survey coverage for onchocerciasis elimination in selected endemic areas of Ethiopia.
(DOCX)

## Abbreviations

APOC: African Program for Onchocerciasis Control
CDD: Community Drug Distributors
CDTi: community-directed Treatment with Ivermectin
CI: Confidence Interval
DOTS: Directly Observed Treatment Strategy
ESPEN: Expanded Special Project for the Elimination of Neglected Tropical Diseases
FMOH: Federal Ministry of Health
HDA: Health Development Army
HEWs: Health Extension Workers
MDA: Mass Drug Administration
NTDs: Neglected Tropical Diseases
OCP: Onchocerciasis Control Program
RTI: Research Triangle Institute international
SNNPR: South Nations and Nationalities Peoples Region
TCC: The Carter Centre
USAID: U.S. Agency for International Development
WHO: World Health Organization
