# Supplementary material for: Evaluation of mass treatment with ivermectin program reach and survey coverage for onchocerciasis elimination in selected endemic areas of Ethiopia
Source: PLoS One. 2022 Jul 28;17(7):e0271518. doi: 10.1371/journal.pone.0271518 (PMC9333289; doi:10.1371/journal.pone.0271518)
Supplement: S1 Table — This is the English version interview guide that was used to collect the socio-demographic profile of the study participants and other relevant data to evaluate mass treatment with ivermectin program reach and survey coverage for onchocerciasis elimination in selected endemic areas of Ethiopia. (DOCX) [file pone.0271518.s001.docx]

**S1 Table. English version of the interview guide to evaluate mass treatment with ivermectin program reach and survey coverage for onchocerciasis elimination in selected endemic areas of Ethiopia.**

Interviewee Code________ District__________Kebele__________Village___________

| S. N | Question | Code | Remark |
| --- | --- | --- | --- |
|  | Gender | 1. Male 2. Female |  |
|  | Age | ____________ |  |
|  | Ethnicity | ____________ |  |
|  | Religion | 1. Orthodox  2. Muslim  3. Protestant  4. Catholic  4. other ____________ |  |
|  | Marital status | 1. Single  2. Married  3. Divorced  4. Widowed |  |
|  | Educational background | ____________ |  |
|  | Occupation | 1. Farmer  2. Merchant  3. Civil servant  4. Daily worker  5. Student  6. Other (mention) _______ |  |
|  | Monthly income (ET Birr) | ___________________ |  |
|  | Household size | ___________________ |  |
|  | Years of stayed in the village | ___________________ |  |
|  | Had you been offered ivermectin (show the tablet in your hand) in the May 2017 MDA campaign? | 1. Yes 2. No | If your answer is Yes, proceed to question number 13 |
|  | If your answer is NO, what was the reason for not offering the drug? | 1. Absent during the campaign 2. Didn’t hear about MDA campaign 3. CDD did not come home 4. Pregnant 5. Breast feeding 6. Underage (<5 year) 7. Too old 8. Drug stock out 9. Sick 10. Drug not working 11. Bored taking the drug 12. Other mention)________ |  |
|  | If your answer is YES, have you swallowed the drug offered? | 1. Yes 2. No |  |
|  | If your answer is NO, what was the reason for not swallowing the tablet? | 1. Healthy (not sick) 2. Fear of drug side effect 3. Drug not working 4. Bored swallowing the drug 5. Bad taste 6. Not enough information given 7. Other (mention)_______ |  |
