# Supplementary material for: Evaluation of mass treatment with ivermectin program reach and survey coverage for onchocerciasis elimination in selected endemic areas of Ethiopia
Source: PLoS One. 2022 Jul 28;17(7):e0271518. doi: 10.1371/journal.pone.0271518 (PMC9333289; doi:10.1371/journal.pone.0271518)
Supplement: S2 Table — This is the Amharic translated version of interview guide that was used to collect the socio-demographic profile of the study participants and other relevant data to evaluate mass treatment with ivermectin program reach and survey coverage for onchocerciasis elimination in selected endemic areas of Ethiopia. (DOCX) [file pone.0271518.s002.docx]

**S2 Table. Amharic translated version of the interview guide to evaluate mass treatment with ivermectin program reach and survey coverage for onchocerciasis elimination in selected endemic areas of Ethiopia.**

የተሳታፊ ኮድቁጥር፡________________ወረዳ፡________________ቀበሌ፡_____________________ጎጥ፡___________________

| ቁ | **ጥያቄ** | ኮድ | ማሳሰቢያ |
| --- | --- | --- | --- |
|  | ጾታ | 1. ወንድ 2. ሴት |  |
|  | ዕድሜ |  |  |
|  | ብሄር | ---------------------- |  |
|  | ሀይማኖት | 1. ኦርቶዶክስ  2. ሙስሊም  3. ፕሮቴስታንት  4. ካቶሊክ  4. ሌላ ____________ |  |
|  | የጋብቻ ሁኔታ | 1. ያላገባ  2. ያገባ  3. አግብቶ የፈታ  4. በሞት የተለየ/ችበት |  |
|  | የትምህርት ደረጃ | ---------------------- |  |
|  | ሥራ | 1. ገበሬ  2. ነጋዴ  3. የመንግስት ሰራተኛ  4. በቀን ስራ የሚተዳደር  5. ተማሪ  6. ሌላ ካለ ግለፅ_ ____________ |  |
|  | የወር ገቢ መጠን (ብር) | ___________________ |  |
|  | የቤተሰብ ብዛት | ___________________ |  |
|  | በጎጡ የኖርክበት ዓመት ብዛት | ___________________ |  |
|  | በቅርብ (ባለፈው ግንቦት ወር) በተሰራጨው የአይቨርመክቲን መድሀኒት (በእጅህ ያለው የመድሀኒት ምስል አሳይ) ወስደሃል/ሻል? | 1. አዎ 2. አልወሰድኩም | መልስህ/ሽ አዎ  ወደ ተራ ቁጥር 13 ቀጥል |
|  | መልስህ/ሽ አልወሰድኩም ከሆነ፡ ያልወሰድክበት ምክንያት ምን ነበር? | 1. መድሀኒቱ ሲከፋፈል ሰላልነበርኩ 2. መድሀኒቱ ሲከፋፈል ሰላልተነገረኝ/ሰላልሰማሁ 3. የመደሀኒት አከፋፋይ ወደ ቤቴ ስላልመጣች 4. እርጉዝ ስለነበርኩ 5. እያጠባሁ ስለነበርኩ 6. ከዕድሜ በታች ስለነበርኩ (<5 አመት) 7. በጣም አሮጌ ስለሆንኩ 8. መድሀኒቱ ስላለቀ 9. ታምሜ ስለነበርኩ 10. መድሀኒቱ ስለማይሰራ 11. መድሀኒቱ መውሰድ ስለሰለችኝ 12. ሌላ ግለፅ____________________ |  |
|  | መልስህ/ሽ አዎ ወስጄለሁኝ ከሆነ፡ የተቀበልከው መድሃኒት ውጠውሃል/ሸዋል? | 1. አዎ 2. አልዋጥኩትም |  |
|  | መልስህ/ሽ አልዋጥኩትም ከሆነ፡ ያልዋጥክበት ምክንያት ምን ነበር? | 1. ጤነኛ ስላሆንኩ 2. የመድሀኒቱ ጎጂነት ስለምፈራ 3. መድሀኒቱ ስለማይሰራ 4. መድሀኒቱ መዋጥ ስለሰለችኝ 5. የመድሀኒቱ ጣእም ስለማልወደው 6. ሰለ መድሃኒቱ በቂ መረጃ ስለሌኝ 7. ሌላ ግለፅ___________________ |  |
